# Epigenetic Regulation during Fetal Femur Development: DNA Methylation Matters

**DOI:** 10.1371/journal.pone.0054957

**Published:** 2013-01-28

**Authors:** María C. de Andrés, Emmajayne Kingham, Kei Imagawa, Antonio Gonzalez, Helmtrud I. Roach, David I. Wilson, Richard O. C. Oreffo

**Affiliations:** 1 Bone and Joint Research Group, University of Southampton, Southampton, United Kingdom; 2 Centre for Human Development, Stem Cells and Regeneration Human Development and Health, Institute of Developmental Sciences, University of Southampton, Southampton, United Kingdom; 3 Instituto de Investigación Sanitaria-Hospital Clínico Universitario de Santiago, Santiago de Compostela, Spain; 4 Tohoku University School of Medicine, Sendai, Japan; 5 Stem Cell Unit, Department of Anatomy, College of Medicine, King Saud University, Riyadh, Kingdom of Saudi Arabia; University of Minho, Portugal

## Abstract

Epigenetic modifications are heritable changes in gene expression without changes in DNA sequence. DNA methylation has been implicated in the control of several cellular processes including differentiation, gene regulation, development, genomic imprinting and X-chromosome inactivation. Methylated cytosine residues at CpG dinucleotides are commonly associated with gene repression; conversely, strategic loss of methylation during development could lead to activation of lineage-specific genes. Evidence is emerging that bone development and growth are programmed; although, interestingly, bone is constantly remodelled throughout life. Using human embryonic stem cells, human fetal bone cells (HFBCs), adult chondrocytes and STRO-1^+^ marrow stromal cells from human bone marrow, we have examined a spectrum of developmental stages of femur development and the role of DNA methylation therein. Using pyrosequencing methodology we analysed the status of methylation of genes implicated in bone biology; furthermore, we correlated these methylation levels with gene expression levels using qRT-PCR and protein distribution during fetal development evaluated using immunohistochemistry. We found that during fetal femur development DNA methylation inversely correlates with expression of genes including *iNOS* (*NOS2*) and *COL9A1,* but not catabolic genes including *MMP13* and *IL1B*. Furthermore, significant demethylation was evident in the osteocalcin promoter between the fetal and adult developmental stages. Increased *TET1* expression and decreased expression of DNA (cytosine-5-)-methyltransferase 1 (*DNMT1*) in adult chondrocytes compared to HFBCs could contribute to the loss of methylation observed during fetal development. HFBC multipotency confirms these cells to be an ideal developmental system for investigation of DNA methylation regulation. In conclusion, these findings demonstrate the role of epigenetic regulation, specifically DNA methylation, in bone development, informing and opening new possibilities in development of strategies for bone repair/tissue engineering.

## Introduction

Epigenetic regulation of gene expression is an important mechanism implicated in cell stemness, differentiation and function. Epigenetic modifications are heritable changes in gene expression that are not encoded by the DNA sequence. Methylated cytosine residues at CpG dinucleotides are commonly associated with gene repression [1]. In prokaryotes, the major role of DNA methylation is to protect host DNA against degradation by restriction enzymes. In contrast, eukaryote DNA methylation has been implicated in the control of several cellular processes including differentiation, gene regulation, development, genomic imprinting, X-chromosome inactivation and suppression of repetitive element transcription and transposition [2], [3], [4].

Current research has focused on the role of epigenetic processes in fetal programming and the potential consequences, including enhanced susceptibility, to chronic diseases in adulthood [5], [6]. Strategic loss of methyl groups during development could lead to activation of specific genes in the appropriate lineage [7]. In addition, some of the up-regulated genes are normally exclusively expressed in terminally differentiated cells [8]. These findings raise the possibility that DNA methylation contributes to silencing of tissue-specific genes in non-expressing cells, confirming DNA methylation to be a global repressor of gene expression [7].

The methylation status of DNA domains appears to be faithfully propagated during development [9]. The DNA methylation maintenance enzyme DNA methyltransferase 1 (DNMT1) is partly responsible for this stability, but there is likely to be another, as yet unknown, component that regulates this process [7]. Furthermore, inhibition of *DNMT1* results in reduced methylation and activation of target genes.

In contrast, in primordial germ cells, the genome undergoes extensive demethylation, including the removal of previous parent-specific methylation marks regulated by imprinted gene expression [3]. New imprints occur during gametogenesis, in a parent-of-origin-specific manner. Within a few days of fertilization, genome-wide demethylation occurs followed by a wave of *de novo* methylation, both of which are resisted by imprinted loci [10]. Subsequently DNA methylation patterns must then be maintained during the phase of rapid cellular proliferation in fetal and postnatal development.

Here we provide evidence for epigenetic regulation during fetal femur development. Human fetal femurs of the age used in this study contain predominantly epiphyseal chondrocytes surrounded by a perichondrium/periosteum of an outer fibroblastic layer and, an inner mesenchymal stem cell layer with osteogenic, chondrogenic and adipogenic differentiation potential as published by Mirmalek-Sani and coworkers [11]. Such multipotency confirms human fetal bone cells (HFBCs) to be an ideal developmental system for investigation of DNA methylation regulation. In order to explore a potential link between DNA methylation changes in gene expression observed during fetal development, we have selected genes that we have previously reported to be associated with osteoarthritis (OA) [12], [13], [14]. Using human embryonic stem cells (hESCs), HFBCs, adult chondrocytes and a STRO-1^+^ skeletal stem cell containing population of adult bone marrow, we have examined a spectrum of developmental stages of femur development.

## Materials and Methods

### Fetal Sample Procurement

Human fetal femurs were obtained after termination of pregnancy according to guidelines issued by the Polkinghome Report and with ethical approval from the Southampton & South West Hampshire Local Research Ethics Committee. Fetal age was determined by measuring fetal foot length and expressed in weeks post conception (WPC). In total 12 samples were used (cultured and uncultured) with a mean age of 8.3±1.0 WPC. Skeletal muscle surrounding the femur was removed in sterile phosphate-buffered saline (PBS) prior to femur dissection and digestion with collagenase B overnight. The cell suspension was filtered (70 µm filter) and collected cells were either directly lysed for nucleic acid isolation or cultured on tissue culture plastic in α-MEM containing 10% FCS.

### Cartilage Procurement and Chondrocyte Isolation

Adult femoral heads were obtained with informed patient consent and the permission of the Local Ethics Committee following joint replacement surgery due to OA (n = 13, age 71.6±8.2 years; 3–5 OARSI score) or due to fracture of the neck of femur (normal) (n = 15, age 76.8±16.5 years) (used as a non-OA control) [15]. Cartilage was dissected within 6 hours of surgery and chondrocytes from the surface layer of OA femoral heads or the deep zone of normal cartilage were isolated, as in previous studies [15]. The cartilage was cut into small pieces and digested by sequential treatment with 10% trypsin in PBS for 30 minutes; 1 mg/ml of hyaluronidase in PBS for 15 minutes and finally collagenase B in DMEM/F12 for 12–15 hours at 37°C.

### Bone Marrow Procurement and STRO^+^ Isolation

Bone marrow was obtained with informed patient consent and the permission of the Local Ethics Committee following joint replacement surgery. Marrow cells were isolated from trabecular bone by suspending in α-MEM. The STRO^+^ fraction, reported to contain the skeletal/mesenchymal stem cell population and osteoprogenitor cells [16], [17], was isolated by magnetic activated cell sorting as previously described [18] using STRO-1 antibody hybridoma supernatant (hybridoma cell line was a kind gift from Dr J Beresford, University of Bath). The STRO^+^ and STRO^-^ fractions were collected and RNA/DNA isolated immediately (uncultured) or incubated on tissue culture plastic in basal media (10% FBS, α-MEM) at 37°C in a humidified incubator, 5% CO_2_.

### Human Embryonic Stem Cell Culture

Hues-7 human embryonic stem cells (hESCs) (D. Melton, Howard Hughes Medical Institute/Harvard University) were initially cultured on γ-irradiated mouse embryonic fibroblasts (MEFs) in Knockout DMEM (Invitrogen) supplemented with 10% knockout serum replacement (Invitrogen), 1 mM L-glutamine (Invitrogen), 50 µM β-mercaptoethanol (Sigma), 0.1 mM non-essential amino acids (Invitrogen), 10 ng/ml basic FGF (Peprotech Ltd, London, UK) and 100 µg/ml penicillin/streptomycin (Invitrogen). Subsequent maintenance of hESCs on matrigel coated (BD Biosciences) tissue culture plastic with 24 hours MEF-conditioned medium (C.M.) followed. Throughout, hESCs were incubated at 37°C in atmospheric (∼20%) oxygen. RNA and DNA were isolated from hESCs following a minimum of 3 passages on matrigel coated tissue culture plastic.

### DNA and RNA Extraction

Total RNA and genomic DNA were isolated simultaneously using Qiagen AllPrep DNA/RNA Mini Kit according to the manufacturer’s instructions. RNA was reverse-transcribed with avian myeloblastosis virus reverse transcriptase, oligo(dT)_15_ and random primers [19].

### Quantitative Reverse Transcription-polymerase Chain Reaction (qRT-PCR)

qRT-PCR was conducted using Power SYBR Green PCR Master Mix (Applied Biosystems) and ABI Prism 7500 detection system (Applied Biosystems). Exon-exon boundary primers were designed with Primer Express 3.0 software (Applied Biosystems) with the exception of IL1B, which was designed by and purchased from PrimerDesign Ltd, Southampton, UK. Primer sequences used were iNOS GAGGAGCAGGTCGAGGACTAT (F), TCTTCGCCTCGTAAGGAAATAC (R); COL9A1 CCTGGTGCTCTTGGTTTGA (F), CACGCTCCCCCTTTTCTC (R); MMP13 TTAAGGAGCATGGCGACTTCT (F), CCCAGGAGGAAAAGCATGAG (R); IL1B TGGCAATGAGGATGACTTGTTC (F), CTGTAGTGGTGGTCGGAGATT (R); osteocalcin GGCAGCGAGGTAGTGAAGAG (F), CTCACACACCTCCCTCCT (R); DNMT1 CAGGCCCAATGAGACTGACA (F), GTGGGTGTTCTCAGGCCTGTAG (R) and GAPDH CCAGGTGGTCTCCTCTGACTTC (F), TCATACCAGGAAATGAGCTTGACA (R). Samples were run in triplicate, Ct values were analysed using the 2^−ΔΔCt^ method and the expression of target genes were normalised to GAPDH house keeping gene expression.

### Analysis of DNA Methylation by Pyrosequencing

500 ng genomic DNA was bisulfite treated using EZ DNA Methylation-Gold™ Kit (Zymo Research Corporation) according to the manufacturer’s instructions. Promoter regions of interest were amplified using Platinum® PCR Normal Supermix or High Fidelity (Invitrogen) and purity confirmed by agarose gel electrophoresis. Percentage DNA methylation in the promoter regions was quantified using pyrosequencing primers designed with Pyrosequencing™ Assay Design Software Ver 2.0 (Qiagen) and PyroMark™ MD (Qiagen) according to the manufacturer’s instructions. Primer sequences used were iNOS TTGGATGGTATGGGGTGAGTATAAAT (F), CCAATCCCCTCATCAAAAATAACC (R), TTTTAAAACAAAAATAAAACTAAATTCTCT (Seq); COL9A1_1 GTTGTTGTGAGAATTAAATGGTATTAAG (F), ACACCCAACAATCATTATTTATCA (R), CCCAACAATCATTATTTATC (Seq); COL9A1_2 AGGGATTGAAATTTAGGTTGAT (F), AAATTCCAATAAAAATATACCCACTAA (R), GGATTGAAATTTAGGTTGAT (Seq); COL9A1_3 TGAGGGTTAAAAGTAAAGGGAGAG (F), TTTCCCCTATAAATCCCTCCTT (R), GGGAGAGAATTAGAGGTATT (Seq); MMP13_1 AATTAGTATTAAGTTTTTTTTTATGGAAGT (F), TTCAACAAAATCTCAAAACCCATCTAA (R), AAATTTTTTTTTTTTTACCTTCTAT (Seq1), CTCAAAACCCATCTAAC (Seq2); MMP13_2 ATGGGTTTTGAGATTTTG (F), ACCCCTAAATACATCTTAAATA (R), CAATCACTTAAAAATAAACATACTT (Seq1), AATAATACCTAAAAACTATTATC (Seq2); IL1B_1 ATGGAAGGGTAAGGAGTAGTAA (F), CCCACATATACTAAATTTAAACATTCTT (R), ATACTAAATTTAAACATTCTTCTA (Seq); IL1B_2 ATGAAGATTGGTTGAAGAGAATTTTAGA (F), ATTTCTCAACCTCCTACTTCTACTTTTAA (R), ATTTTAGAGTAGTTTGTTGTG (Seq); Osteocalcin_1 AGTAGGTTGTTTTTGGTGATTTAT (F), CCAACTATCTCACAACCTATAATTTC (R), GGTTGTTTTTGGTGATTTA (Seq); Osteocalcin_2 AGGTAGTTTGTTGTGGGTGTAGTT (F), CCCCACCTCCATTAACTTTAA (R), GTTTGTTGTGGGTGTAGT (Seq).

### Immunohistochemistry

Whole femurs were fixed in 95% ethanol, embedded in paraffin wax and sectioned at 6 µm. Permeabilisation with 0.1% Triton X-100 and pronase enzymatic digestion for COL9A1 were employed. Endogenous peroxidase activity was quenched with 3% H_2_O_2_, sections were blocked with 1% bovine serum albumin (BSA) in PBS and incubated overnight at 4°C with primary antibody in 1% BSA (iNOS (BML-SA200, Enzo Life Sciences, Inc., PA), COL9A1 (ab75807, Abcam, UK), MMP13 (ab39012, Abcam, UK), IL1B (AF-201-NA, R&D Systems, Inc., MN) and STRO-1 (hybridoma supernatant, Dr J Beresford, University of Bath). Sections were washed with PBS and incubated for 1 hour with biotinylated secondary antibody. Avidin-biotin method linked to peroxidase and 3-amino-9-ethylcarbazole (AEC) and counterstaining with 1% alcian blue was used to visualize, positive staining appears reddish-brown. Appropriate isotype and secondary antibody only controls were used to confirm specific staining. Virtual digital slides were acquired using a dotSlide system (Olympus) and analysed with OlyVIA software (2.4).

### Statistical Analysis

Data is expressed as the mean ± SD. Statistical analysis was performed using SPSS software, version 17.0 (SPSS, Chicago, IL) where Mann-Whitney 2-tailed U test was used to compare between groups or ANOVA where it was necessary, and Spearman’s rank correlation coefficient was used for correlation studies. *P* values less than 0.05 were considered significant.

## Results

### *iNOS* Gene Expression Correlates with Loss of Methylation during Fetal Femur Development

**Figure 1:**
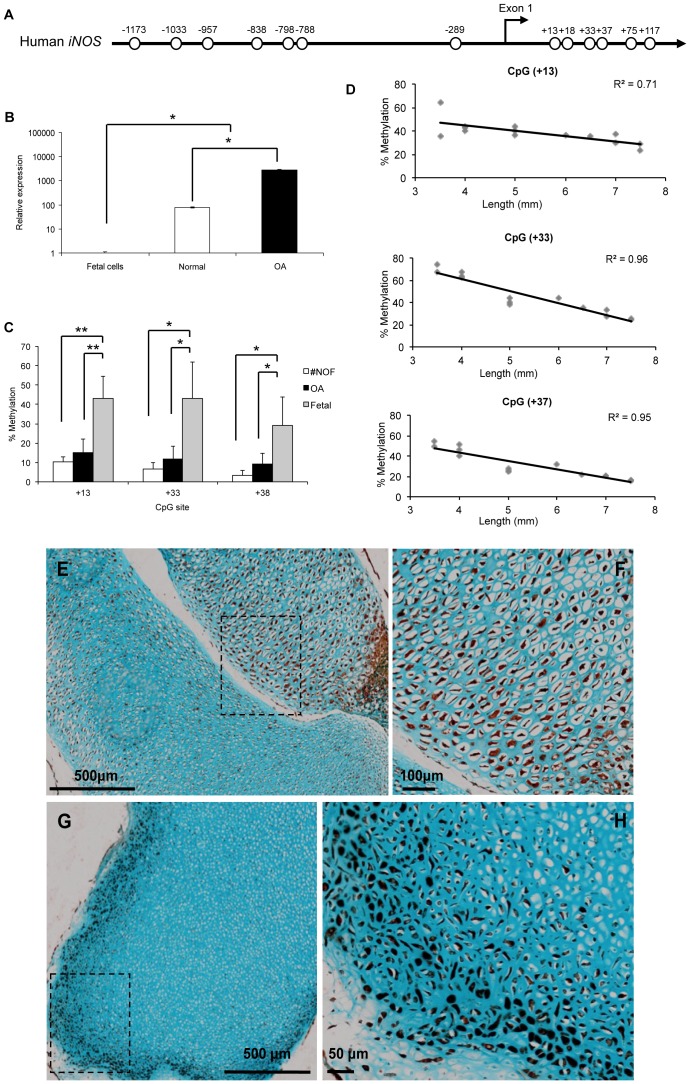
*iNOS* epigenetic regulation during fetal femur development. A . Human *iNOS* proximal promoter, each circle represents a CpG site. **B**. Relative expression of *iNOS* in HFBCs cells and adult chondrocytes from healthy and OA patients. **C**. Methylation status of CpG sites localised at the transcription start site of *iNOS* gene in HFBCs and adult chondrocytes. **D**. Correlation between foot length and CpG sites methylation level. **E**. Localization of iNOS in a fetal bone from a 4.5 mm fetal sample. See a higher magnification of the square in **F**. **G**. Immunohistochemical localization of iNOS in a fetal bone from an 8.5 mm fetal sample. See a higher magnification of the square in **H**.

The human *iNOS* promoter (sparse CpG promoter) has 7 CpG sites within the 1,400-bp sequence upstream of exon 1 and 6 CpG sites within the exon 1 (GenBank accession No. AF017634) (Figure 1A); the transcription start site was located 30 bp downstream of a TATA box [20]. In activated chondrocytes, *iNOS* expression is known to be significantly higher than in healthy chondrocytes [21]. However, a significant increase in *iNOS* expression from HFBCs to adult healthy chondrocytes was observed (Figure 1B). This significant increase correlated with a significant loss of methylation of CpG sites localized near the transcription start site of *iNOS* gene. The CpG sites in the initiation region of the first exon (+13, +33 and +37), were largely unmethylated in normal and OA patients but, critically, increased methylation was observed in HFBCs (Figure 1C). Furthermore, the degree of methylation was inversely correlated to foot length and, thus, to the age of the sample (CpG sites +13, +33 and +37 mean R = 0.87) (Figure 1D). However, immunohistochemical analysis demonstrates that the majority of cells from fetal femurs produce iNOS (Figure 1E–H). Although iNOS distribution was observed to change with the age of the sample; in tissue samples from 7–8 WPC fetus (n = 8), iNOS displayed a tissue wide distribution (Figure 1E–F). However, in samples from 8.5–9 WPC fetus (n = 6), iNOS staining was enhanced with localisation limited to the superficial area (Figure 1G–H).

### *COL9A1* Gene Expression Correlates with Loss of Methylation during Fetal Femur Development

**Figure 2:**
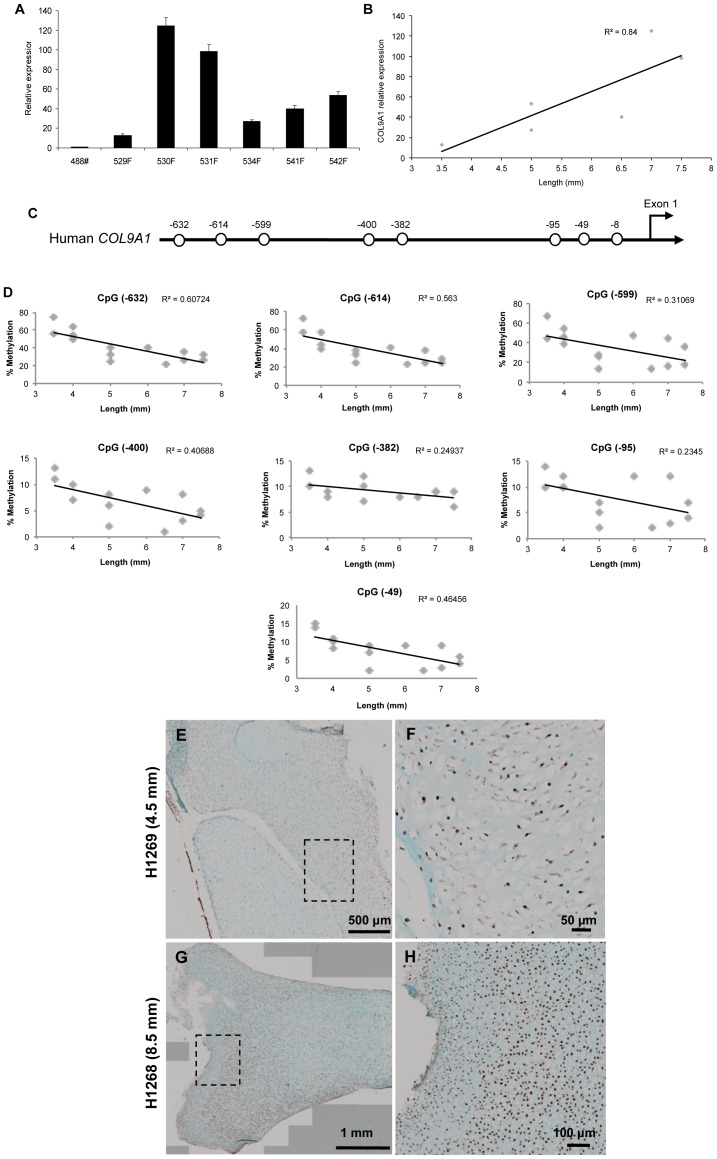
*COL9A1* regulation during fetal femur development. A . Relative expression of *COL9A1* in different HFBCs samples. **B**. Correlation between foot length and *COL9A1* relative expression. **C**. Human *COL9A1* proximal promoter, each circle represents a CpG site. **D**. Correlation between foot length and the percentage of methylation of all the CpG sites localised at the proximal promoter. **E**. Immunohistochemical localisation of COL9A1 in a fetal bone from a 4.5 mm fetal sample. See higher magnification in **F**. **G**. Localisation of COL9A1 in a fetal bone from an 8.5 mm fetal sample. See a higher magnification of the square in **H**.

High levels of *COL9A1* expression were found in all the HFBCs samples analysed (59.40±39.55, n = 6) (Figure 2A). *COL9A1* expression levels were significantly correlated with fetal foot length (R = 0.84) (Figure 2B).

The human *COL9A1* promoter (sparse CpG promoter) contains 8 CpG sites in the 1,000-bp sequence upstream of exon 1 (GenBank accession No. AF036110), relative to the transcriptional start site (+1) [22] (Figure 2C). In general, methylation levels in all CpG sites in the *COL9A1* promoter region were inversely correlated with foot length and developmental age; i.e. the oldest fetal sample analysed displayed the lowest percentage of CpG site methylation (Figure 2D).

Immunohistochemical analysis showed that the majority of cells from fetal femurs stained positively for COL9A1 (Figure 2E–H). Indeed, COL9A1 distribution was observed to change with the age of the sample; in tissue samples from 7–8 WPC fetus, COL9A1 staining was weak with widespread distribution throughout the tissue (Figure 2E–F). In contrast, in older samples from 8.5–9 WPC fetus, COL9A1 staining was considerably stronger and was typically localised to the proximal and distal heads (Figure 2G–H) of the fetal femur.

### *MMP13* Gene Expression does not Correlate with Loss of Methylation during Fetal Femur Development

**Figure 3:**
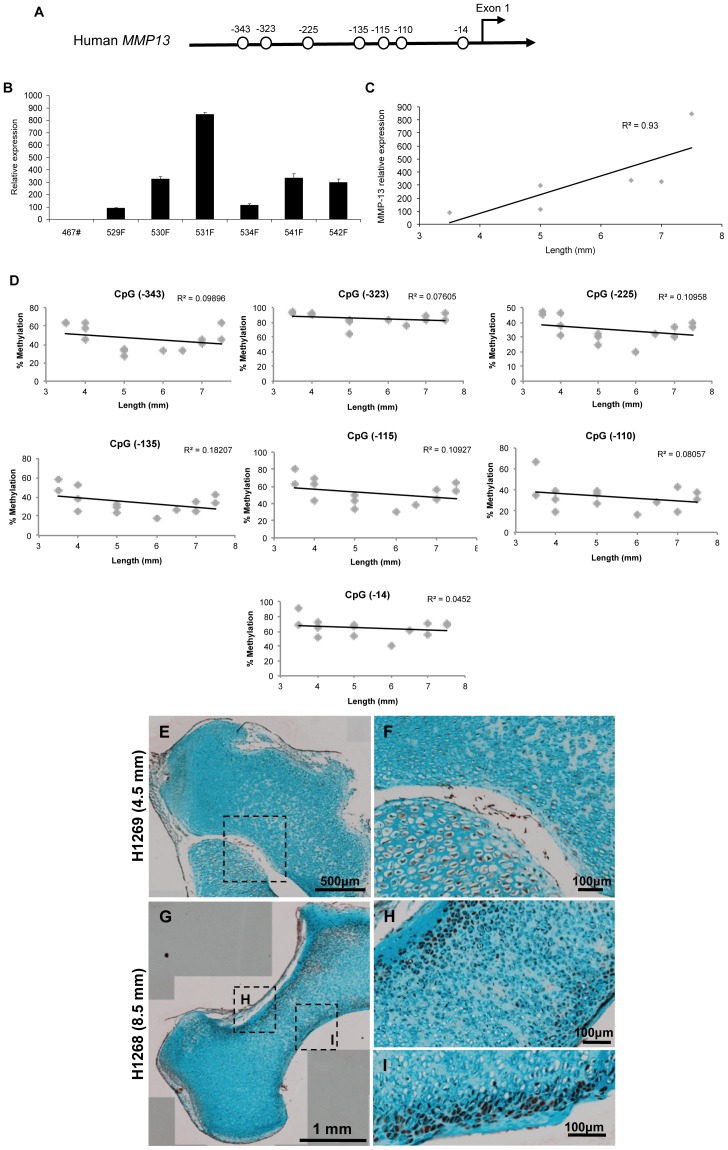
Changes in *MMP13* expression during fetal femur development. A . Human *MMP13* proximal promoter, each circle represents a CpG site. **B**. Relative expression of *MMP13* in different HFBCs samples. **C**. Correlation between sample age and *MMP13* relative expression. **D**. Correlation between foot length and the percentage of methylation of all the CpG sites localised at the proximal promoter. **E**. Immunohistochemical localisation of MMP13 in a fetal bone from a 4.5 mm fetal sample. See higher magnification in **F**. **G**. Localisation of MMP13 in a fetal bone from an 8.5 mm fetal sample. See a higher magnification of the squares in **H** and **I**.

The human *MMP13* promoter (sparse CpG promoter) has 7 CpG sites in the 400-bp sequence upstream of exon 1 (GenBank accession No. NG021404); the transcription start site is located 22-bp upstream of the ATG start codon [23] (Figure 3A). Substantial expression of *MMP13* was detected in HFBCs samples (354.2±254.2, n = 7) (Figure 3B). Indeed, expression significantly correlated with femur length (Figure 3C). However, this significant increase in gene expression is not correlated with loss of methylation in any of the CpG sites inside the promoter (Figure 3D), in contrast to our observations for *iNOS* and *COL9A1*.

Immunohistochemical analysis indicated that a considerable proportion of cells from fetal femurs produce MMP13 (Figure 3E–I) with MMP13 expression noted to alter with the age of the sample. Thus in a younger tissue sample (from 7–8 WPC), MMP13 staining was typically weak and widespread in distribution across the tissue (Figure 3E–F). However, in older fetal samples (from 8.5–9 WPC), MMP13 staining was significantly stronger and observed to be predominantly distributed in the diaphysial centre, specifically in the distal part of the bone (Figure 3G–I).

### *IL1B* Gene Expression does not Correlate with Loss of Methylation during Fetal Femur Development

**Figure 4:**
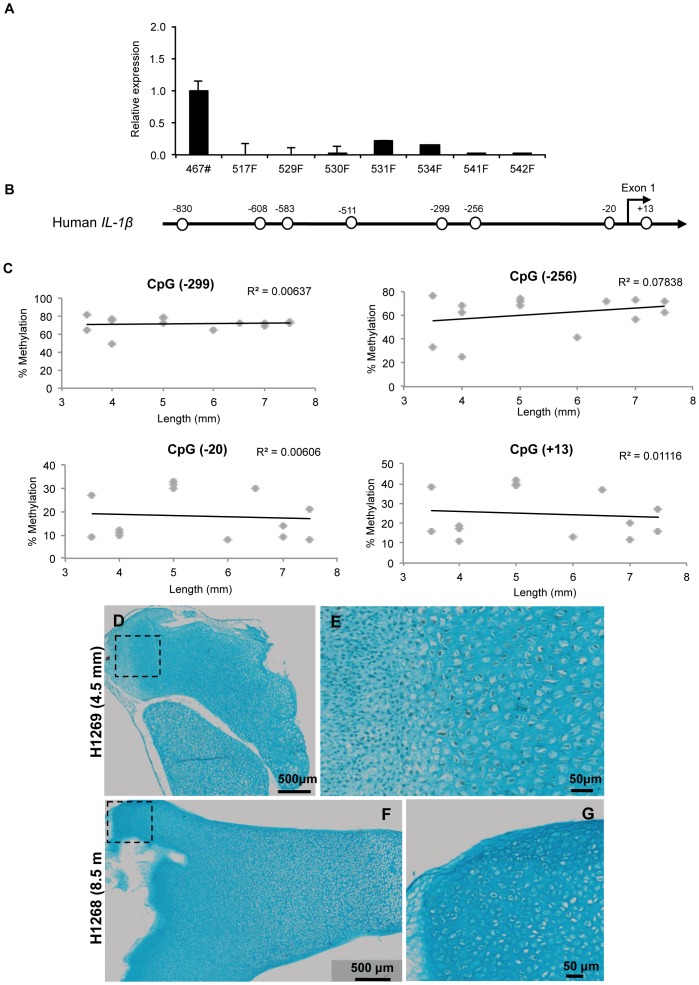
Absence of considerable changes in *IL1B* during fetal femur development. A . Relative expression of *IL1B* in different HFBCs samples compared to healthy chondrocytes. **B**. Human *IL1B* proximal promoter, each circle represents a CpG site. **C**. Correlation between foot length and the percentage of methylation of all the CpG sites localised at the proximal promoter. **D**. Localisation of IL1B in a fetal bone from a 4.5 mm fetal sample. See higher magnification in **E**. **F**. Immunohistochemical localisation of IL1B in a fetal bone from an 8.5 mm fetal sample. See a higher magnification of the square in **G**.

No significant expression of *IL1B* was detected in HFBCs compared with healthy adult chondrocytes (Figure 4A). The human *IL1B* promoter (sparse CpG promoter) has 7 CpG sites in the 900-bp sequence upstream of exon 1 and 1 CpG site within exon 1 (GenBank accession No. AY137079) (Figure 4B); the transcription start site was located 31 bp downstream of a TATA box [24]. CpG sites within this proximal promoter region did not demonstrate a significant correlation between methylation levels and the size of the examined sample (Figure 4C). Interestingly at the CpG site located 299 base pairs upstream of the coding start site, a significant correlation between methylation levels and the size of the fetal femur sample was observed only in uncultured samples (R = 0.80). We have previously reported that this position is a crucial CpG site in the epigenetic induction of *IL1B* in OA chondrocytes [12], [13].

Immunohistochemical analysis showed there was no significant expression of IL1B (Figure 4D–G). IL1B staining was only detectable in tissue samples from 7–8 WPC fetus, although IL1B staining was weak and observed to be distributed throughout the tissue (Figure 4D–E).

### Osteocalcin Promoter Region Methylation

**Figure 5:**
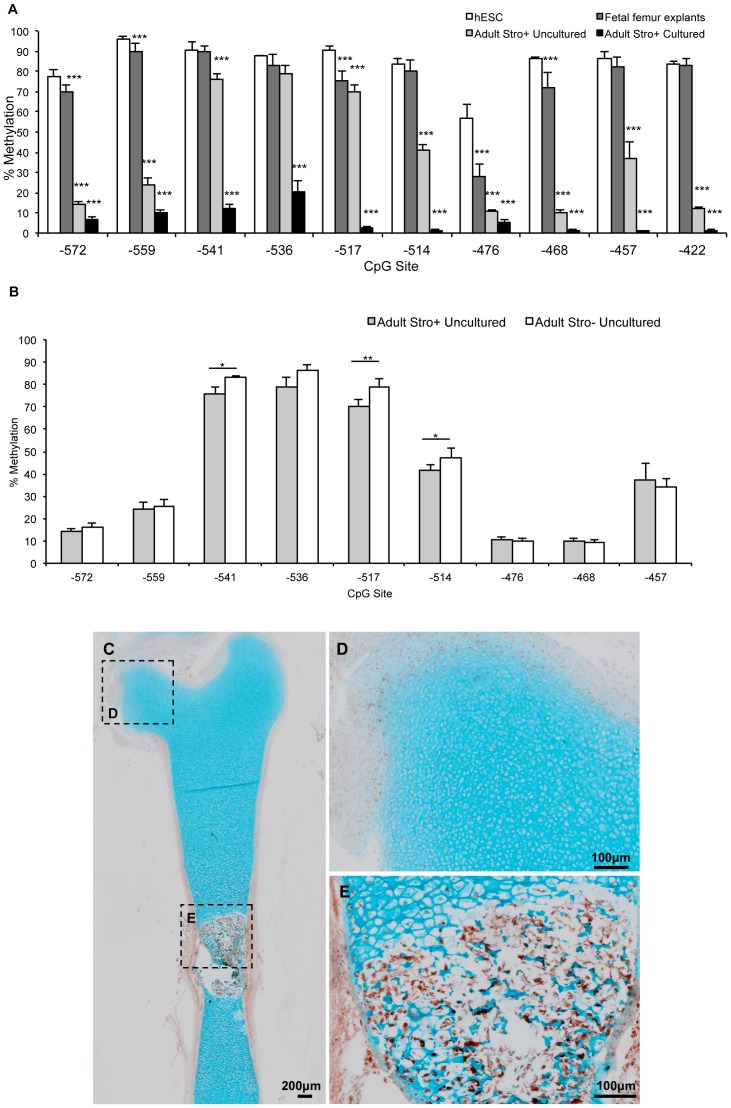
Changes in the methylation status of osteocalcin during fetal femur cell development. **A.** Osteocalcin promoter methylation status in embryonic, fetal and adult cells. **B.** Osteocalcin promoter methylation status in STRO-1+ (skeletal stem cell containing) and STRO-1^−^ fractions of adult bone-marrow cells. **C**. Localisation of STRO-1 in a fetal bone from a 6 mm fetal sample. See higher magnification in **D–E**.

The human Osteocalcin promoter (sparse CpG promoter) has 10 CpG sites within the vitamin D response element [25]. Assessment of methylation status of this region in human embryonic stem cells (hESCs) revealed a hypermethylated state, consistent with a lack of osteocalcin expression (data not shown), and epigenetic silencing via methylation. Similarly, DNA isolated from fetal femur explants was hypermethylated at each CpG site investigated. Previously we have reported osteocalcin expression in HFBCs only when cells are incubated in osteogenic conditions [11]. However, at CpG sites 572, 559, 517 and 468 base pairs upstream of the osteocalcin start site, methylation was significantly lower for fetal femur DNA than hESC DNA (Figure 5A).

Significant demethylation was evident between the fetal and adult developmental stages. Methylation levels of CpG sites 572, 559, 541, 517, 514, 476, 468, 457 and 422 base pairs upstream of the coding start site were statistically lower for uncultured STRO^+^ adult skeletal stem cells compared to HFBCs indicating demethylation at a developmental stage between fetal and adulthood. However, osteocalcin RNA expression was undetectable in STRO^+^ adult skeletal stem cells indicating that demethylation occurs prior to induction of osteocalcin expression. In all instances, culture of STRO^+^ adult skeletal stem cells resulted in further demethylation leading to a hypomethylated state regardless of osteocalcin expression (Figure 5A).

On examination of the STRO^+^ and STRO^-^ fractions, it was observed that three CpG sites (541, 517 and 514 base pairs upstream of the coding start site) displayed significantly greater methylation in STRO^-^ cells (Figure 5B).

Immunohistochemical analysis demonstrated that a considerable proportion of cells from fetal femurs produce STRO-1 (Figure 5C–E). STRO-1 staining was typically weak in the proliferating chondrocyte population with localisation limited to the superficial area (Figure 5D); however, in the hypertrophic chondrocyte population, STRO-1 staining was significantly stronger (Figure 5E).

### Decreased *DNMT1* Expression in Adult Chondrocytes

**Figure 6:**
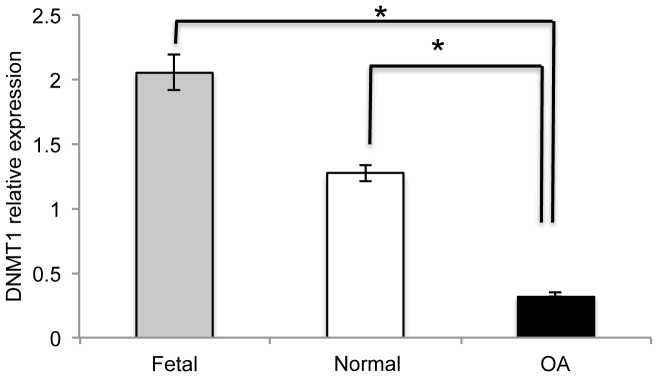
Changes in *DNMT1* expression in HFBCs and adult chondrocytes. Relative expression of *DNMT1* during chondrocyte evolution. Levels of DNMT1 were measured by qRT-PCR.

qRT-PCR was used to determine whether decreased expression of *DNMT1* contributed to the observed loss of DNA methylation in chondrocytes from adult cartilage. *DNMT1* expression in healthy chondrocytes was observed to be almost half that of HFBCs (Figure 6). Additionally, *DNMT1* expression in OA chondrocytes from the surface zone was less than half that observed in control chondrocytes from patients with femoral neck fracture (Figure 6), as it had been previously shown [13]. *DNMT1* expression levels were found to be independent of fetal sample age (R = 0.06).

### TET Proteins in Fetal Cells

**Figure 7:**
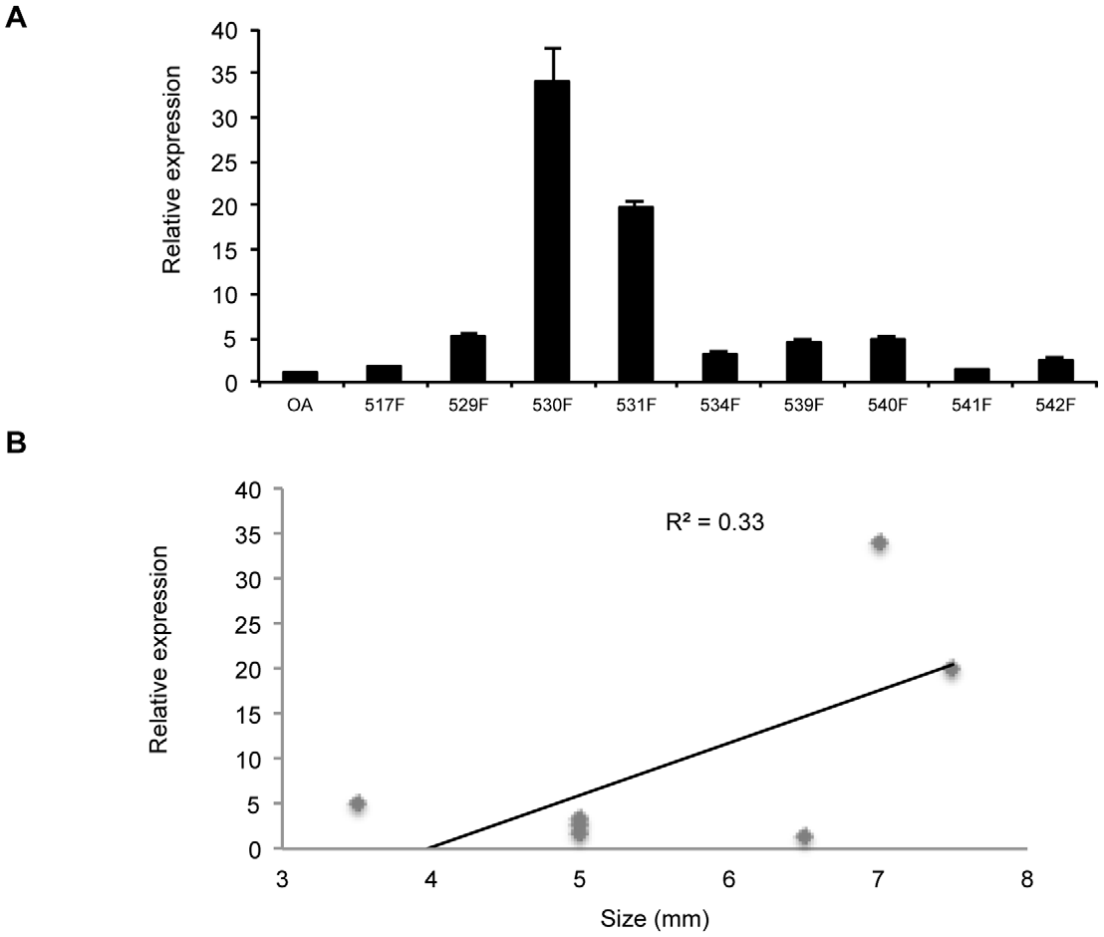
Relative expression of *TET1* in HFBCs cells. **A**. Levels of *TET1* were measured by qRT-PCR in comparison with healthy chondrocytes. **B.** Correlation of *TET1* with sample age.

*TET1-3* expression was analysed by qRT-PCR. However, only *TET1* showed higher expression in HFBCs compared to human adult chondrocytes (3.8±5.0 versus 1.1±0.8; n = 9) (Figure 7A). However this difference was not statistically significant (P = 0.15), and *TET1* expression was observed to display a very weak correlation to fetal age (Figure 7B).

## Discussion

Epigenetic marks, including CpG methylation, are generally stable in somatic cells; however, during at least two developmental time periods, the epigenome undergoes extensive epigenetic programming [26]. These critical windows of development include gametogenesis as well as early pre-implantation embryos [3]. The pattern of distribution of methyl groups in DNA differs depending on cell type and confers a cells specific identity on DNA during cellular differentiation and organogenesis. This is an innate and highly programmed process [27] and an understanding of the underlying epigenetic mechanisms that regulate gene expression in bone development provide important possibilities in tissue repair.

The human fetal femurs used in these studies contain predominantly epiphyseal chondrocytes surrounded by a perichondrium/periosteum consisting of an outer fibroblastic layer and an inner layer of non-committed mesenchymal stem cells, the latter capable of differentiating either along the chondrogenic, osteogenic or adipogenic lineages [11]. A first biological characterization of HFBCs has already been reported indicating that these cells have an enhanced capacity to proliferate compared with human osteoblasts and a higher capacity to differentiate *in vitro* into bone cells compared with mesenchymal cells [28]. Furthermore, other authors suggest that cells taken before 8 weeks of development represent a predominantly more primitive cell population [29]. To test the utility of HFBCs to study the epigenetic mechanisms implicated on bone development, we have examined the influence of DNA de-methylation on gene expression, with emphasis on physiological genes vital for bone biology (*iNOS* and *COL9A1*) and, catabolic/pathologic genes important for bone modulation and remodelling as well (*MMP13* and *IL1B*). These genes, *iNOS, COL9A1, MMP13* and *IL1B* were selected as key genes linked to OA; in this regard we have already published on the importance of IL-1B and MMP13 in the context of cartilage and OA [12], [13], [14], and sought to extend these observations to our understanding during fetal development and the role of epigenetics. In all studies, human fetal femur derived skeletal cells were used directly after digestion as culture conditions alone can induce de-methylation.

iNOS plays an important role in various cell systems including bone, where it has a dual role. Evidence from gene-knockout studies indicates that bone formation and resorption are regulated by NO [30]. Furthermore, a number of studies have indicated the active involvement of NO in bone healing [31], [32], [33], [34], bone development [35], [36], [37], [38], [39] and bone loss [40], [41]. Thus tight regulation of *iNOS* would be expected for this gene, and we observed such an exquisite epigenetic regulation of *iNOS*. The increase in expression levels correlated with loss of methylation in specific CpG sites localized at the transcription start site. Similarly, A2(1) collagen transcription is inhibited by methylation sites within the first exon at the initiation site of transcription [42]. Others have reported that effective gene suppression was observed when promoters were methylated in the pre-initiation domain [43]; methylation of coding regions are common in mammalian genes and reportedly improve genome stability [44]. In the context of bone, Delgado-Calle and collaborators have recently demonstrated that the methylation status of CpG dinucleotides within the proximal promoter plays an important role in the regulation of *SOST* (encodes Sclerostin protein) expression, impairing its expression in osteoblasts and other extraskeletal cells and permitting expression in osteocytes [45].

In HFBCs, in which we observed higher levels of methylation but no mRNA expression, inhibition of DNA methylation by 5-aza-2′-deoxycytidine (5-aza) and trichostatin A (TSA) treatment, was not sufficient to induce *iNOS* expression; similar results were also recorded for adult chondrocytes (data not shown).

Type IX collagen, although a minority molecule in hyaline cartilage, is a key component acting as a molecular bridge between cartilage collagen fibrils and other matrix components, possibly proteoglycans [46], [47]. It has been shown that the lack of collagen IX seriously affects growth plate organization, especially in young animals [48]; furthermore, *COL9A1* deregulation is an accepted marker of OA [49]. Tight regulation is considered to occur during bone development and the current results are consistent with epigenetic control during fetal development. Critically a sequential loss of methylation was noted to correlate with an increase in gene expression. Further experiments were conducted and, after treatment with 5-aza plus TSA, *COL9A1* expression correlated with DNA methylation (Imagawa et al., manuscript in preparation).

Bone development requires a complex remodelling of the extracellular matrix, and this is predominantly mediated by matrix metalloproteinases (MMPs) [30]. MMP13 is a potential target for NO regulation in bone development, with Cbfa-1 (RUNX2) a mediator of NO action [30] **.** We observed a spatial and sequential *MMP13* expression, however this was independent of the methylation status of *MMP13* promoter. Thus it would appear genes involved in normal femur development are epigenetically regulated during fetal femur development, however expression of catabolic genes such as *MMP13* or *IL1B* are independent of DNA methylation. Nevertheless, previous experiments within our group showed interesting results in chondrocytes with 5-aza and TSA capable of inducing *IL1B* expression [13], and *MMP13* (Hashimoto et al., manuscript in preparation); however, in HFBCs no changes in expression were detected for these genes (data not shown).

Furthermore, the reduction in *DNMT1* expression levels observed in adult chondrocytes compared to HFBCs is in agreement with the increment of expression of certain vital genes for bone development.

Recently, it has been demonstrated that oxidation of 5-methylcytosine to 5-hydroxymethylcytosine by TET family hydroxylases may also participate in active DNA demethylation [50], [51]. Specifically, TET1 is involved in embryonic stem cell (ESC) maintenance and inner cell mass (ICM) cell specification, possibly by participating in DNA demethylation [52]. In this regard, we observed lower *TET1* expression levels in adult chondrocytes in comparison to HFBCs samples, where an active de-methylation process is in progress. Conversion of 5-methylcytosine to 5-hydroxymethylcytosine may facilitate passive DNA demethylation by excluding the maintenance DNA methyltransferase DNMT1, which poorly recognizes 5-hydroxymethylcytosine [53]. Interestingly, it has been postulated that the balance between hydroxymetylation and methylation in the genome is inextricably linked with the balance between pluripotency and lineage commitment [54].

Osteocalcin is a non-collagenous protein secreted by osteoblasts and is therefore a specific marker for osteoblast activity [55]. Cells isolated from 12–14 WPC fetal femurs present characteristics of advanced osteoprogenitors compared with human mesenchymal stem cells isolated from bone marrow [29]. The STRO^+^ fraction of adult bone marrow contains a population of skeletal stem cells with osteogenic, chondrogenic and adipogenic differentiation potential [17]. These cells are of potential interest for therapeutic use in bone tissue engineering. It has been reported that reduced DNA methylation of CpG islands in the osteocalcin and osteopontin genes is associated with osteogenic differentiation [56], [57]. We did not detect osteocalcin expression but observed developmental stage-dependent demethylation suggesting that demethylation alone does not regulate osteocalcin expression but that methylation can prevent expression. However, the fact that certain CpG sites displayed significantly greater methylation in STRO^-^ cells appears to be no coincidence given that the STRO^+^ fraction has osteogenic potential and thus the potential to express osteocalcin upon differentiation. Mirmalek-Sani and collaborators demonstrated that HFBCs expressed accelerated osteogenesis evidenced by reduced culture periods and higher levels of bone markers such as RUNX2, alkaline phosphatase (ALP) and COL1A1 compared with human bone marrow derived mesenchymal cells, suggesting that HFBCs are more advanced in their osteogenic program [11]. Montjovent and colleagues also reported enhanced ALP and mineralisation activity compared to adult mesenchymal populations [28]. Interestingly, HFBCs and HMSCs failed to express cartilage markers such as SOX9 and COL2A1. We have performed preliminary experiments using adult human osteosarcoma osteoblast-like bone cell lines (MG63 and Saos-2 unpublished observations). In these studies the human osteoblast like cell lines failed to express our genes of interest. Furthermore, the lack of expression was observed to correlate with high levels of methylation of most CpG sites of these promoters.

To date, there is a paucity of information regarding the role of DNA methylation in the differentiation of adult human skeletal cells, although some recent studies suggest that it is involved in the regulation of a number of bone genes, as well as in bone cell differentiation [56], [58], [59]. Previously, we have shown that monolayer cultures of human bone marrow stromal cells treated for 3 days with the 5-aza or TSA, followed by culture in the absence of modifiers displayed distinct phenotypic changes. 5-aza stimulated osteogenic differentiation (evidenced by enhanced ALP activity, increased Runx-2 expression in monolayer, and increased osteoid formation in 3D cell pellets) whilst pellet cultures of human skeletal cells in chondrogenic media with TSA enhanced cartilage matrix formation and chondrogenic structure [60].

Interestingly, it has recently been published that osteoblasts and osteocytes have opposite DNA methylation profiles in the *ALP* promoter, which is hypomethylated in osteoblasts and hypermethylated in osteocytes, suggesting that DNA methylation is inhibiting *ALP* expression in the latter [61].

In fetal bone femurs, relatively small numbers of differentiated osteoblasts were present along the central diaphysis, as indicated by the presence of the first bone collar. The cells that grew out from the dissected explants during culture could thus theoretically be derived from the cut edges of the epiphysis (early chondrocytes) or from the perichondrium (fibroblastic or mesenchymal stem cells). Given epiphyseal chondrocytes were by far the most frequent cell type present in the fetal femurs and as most outgrowth was observed from the cut edges, it is probable that the starting cells were early chondroprogenitor and mesenchymal progenitor populations [11]. Recent experiments in our group analysed DNA methylation status of genes over- or under-expressed in a sub-set of samples. These studies found that 100% of over-expressing samples but just 43% of under-expressing samples had decreased promoter methylation [62].

### Conclusions

Here we show that the regulation of genes involved during normal bone development are controlled by epigenetic mechanisms, specifically loss of methylation in crucial CpG sites of their proximal promoters. In conclusion, these findings demonstrate the role of epigenetic regulation, specifically DNA methylation, in bone development, informing and opening new possibilities in development of strategies for bone repair and tissue engineering.
